# Supplementary material for: Association between oral microbiome and seven types of cancers in East Asian population: a two-sample Mendelian randomization analysis
Source: Front Mol Biosci. 2023 Nov 21;10:1327893. doi: 10.3389/fmolb.2023.1327893 (PMC10702768; doi:10.3389/fmolb.2023.1327893)
Supplement: Supplementary file 1 [file DataSheet1.ZIP › Supplementary Materials/MR plots for tongue/tongue═╝/Gastric cancer/pheno.1960_to_gastric cancer_forest.pdf]

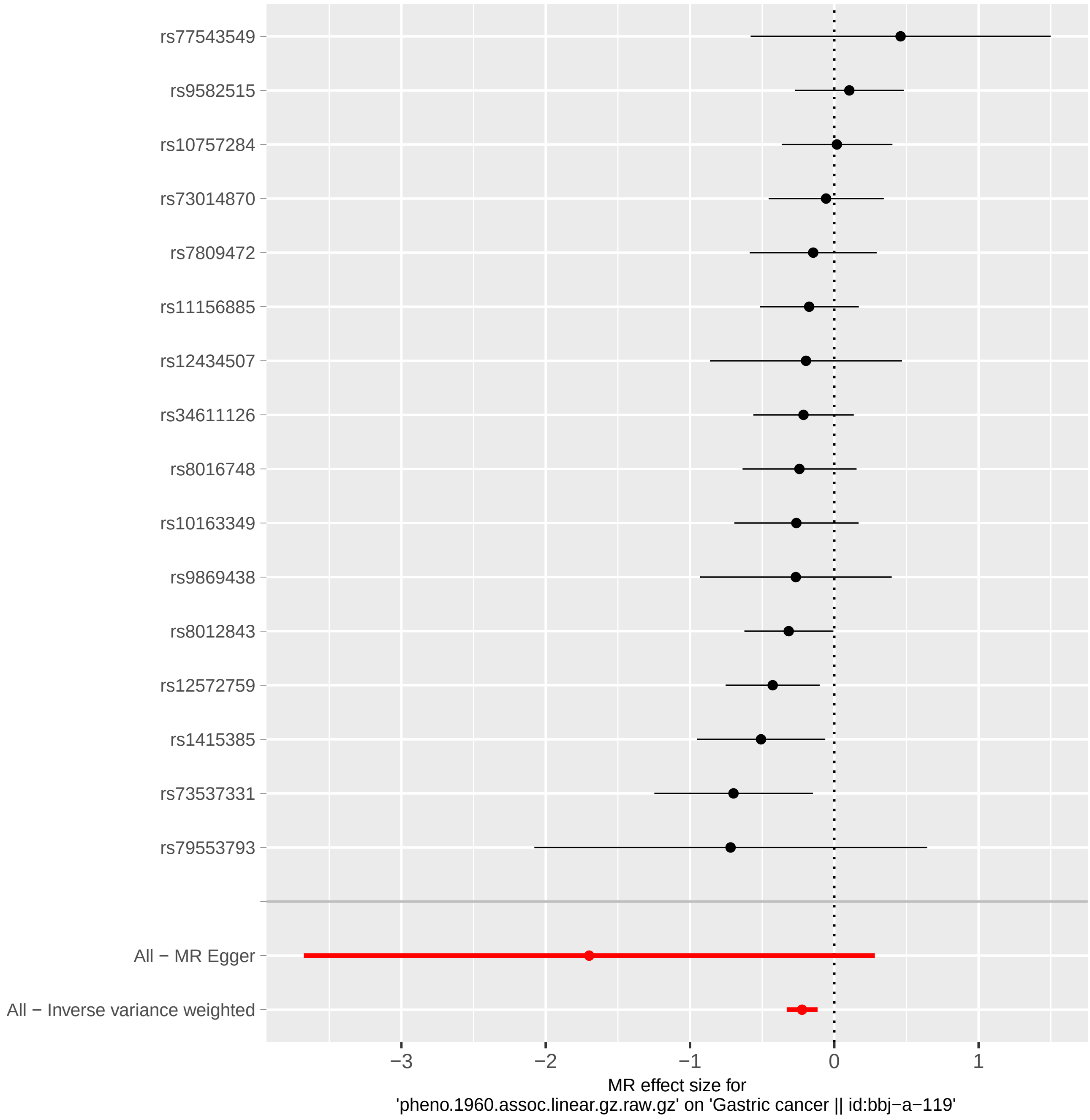
rs77543549
rs9582515
rs10757284
rs73014870
rs7809472
rs11156885
rs12434507
rs34611126
rs8016748
rs10163349
rs9869438
rs8012843
rs12572759
rs1415385
rs73537331
rs79553793
All − MR Egger
All − Inverse variance weighted
−3
−2
−1
0
1
MR effect size for
'pheno.1960.assoc.linear.gz.raw.gz' on 'Gastric cancer || id:bbj−a−119'
